# Apparent pacemaker dysfunction during peptide receptor radionuclide therapy for neuroendocrine tumor

**DOI:** 10.1002/ccr3.1271

**Published:** 2017-12-14

**Authors:** Stefan Asbach, Fabienne Schluermann, Juri Ruf, Christoph Bode, Corinna Lang

**Affiliations:** ^1^ Cardiology and Angiology I Heart Center Freiburg University Freiburg Germany; ^2^ Medizinische Klinik I Hegau‐Bodensee Klinikum Singen Germany; ^3^ Department of Nuclear Medicine Faculty of Medicine University of Freiburg Freiburg Germany

**Keywords:** Exit block, hyperkalemia, pacemaker, radionuclide therapy

## Abstract

This case is a reminder not to overlook rare causes of electrolyte shifts, which may cause reversible changes in pacemaker pacing thresholds.

Peptide receptor radionuclide therapy with radiolabeled somatostatin analogues is an established treatment modality of metastasized irresistible well‐differentiated neuroendocrine tumors. By specific binding to the overexpressed somatostatin receptors of the tumor cells, a tumor‐selective irradiation therapy is possible after i.v. administration of the radioligands (usually coupled to a beta‐emitter such as Lutetium 177) 1.

To minimize radiation exposure to the kidneys, due to tubular reabsorption of the radiopeptides, positively charged amino acids are co‐infused for nephroprotection. However, although a lowering of consecutive kidney radiation dose is achieved, shifts in electrolyte balance, especially significant hyperkalemia, may occur as a side effect of this measure 2.

We present the case of a 69‐year‐old female patient who was treated for metastatic (bone, liver, lymph nodes) atypical carcinoid of the lung with 7.4 GBq ^177^Lu‐DOTATATE and co‐administration of Lysin/Arginin‐amino acid solution. She presented with moderately impaired renal function (GFR 40 mL/min/m^2^) and had received a dual chamber pacemaker for sick‐sinus syndrome three years earlier (Biotronik Entovis DR Berlin, Germany). Otherwise, she did not have any other known cardiovascular disease. Her medical treatment included metformin, torasemid, ramipril, amiodarone, simvastatin, and thyroxin.

After completion of infusion‐therapy on day 1, she suffered recurrent episodes of symptomatic bradycardia, and had to transiently be transferred to the internal medicine ICU for further surveillance. ECG (Fig. 1) was judged to be exit block of the ventricular pacing lead, and after discharge from the nuclear medicine ward, the patient was referred for lead revision. Serum potassium at the time of bradycardia was elevated at 6.1 mmol/L.

**Figure 1:**
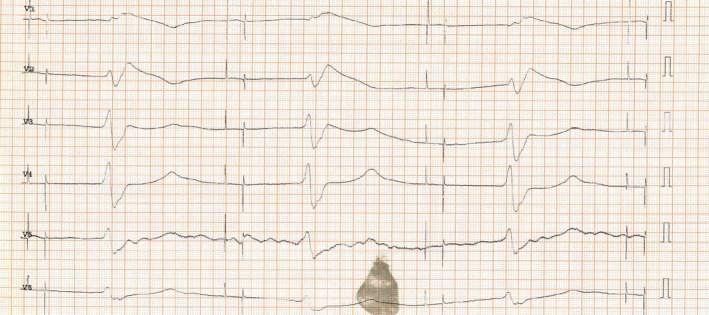
ECG shows atrial and ventricular pacing artifacts, each followed by a ventricular depolarization, constantly coupled but with a latency of 600 ms. Atrial depolarizations are indiscernible. The QRS complex is widened, with a QRS duration of 240 ms, and a Brugada‐like pattern with ST‐segment elevation and T‐wave inversion in leads V1 and V2.

Device interrogation revealed a latency of 600 ms from ventricular pacing stimulus to depolarization, however with constant coupling of the stimulus and the QRS complex (First‐degree pacemaker exit block of the ventricular lead). The atrial lead showed complete exit‐block, intrinsic heart rate during device interrogation was <25 bpm with a different QRS morphology (Fig. 2). Sensing of the delayed ventricular depolarization resulted in reduction in the effective pacing rate to 35 bpm (Fig. 3). A P‐wave was indiscernible, and the paced QRS complex was wide (240 ms) with a Brugada‐like pattern with ST‐segment elevation and T‐wave inversion in leads V1 and V2. After normalization of serum potassium levels, ECG features returned to normal with a ventricular pacing threshold of 0.8 mV@0.5 ms. Ventricular stimulation was immediately followed by depolarization.

**Figure 2:**
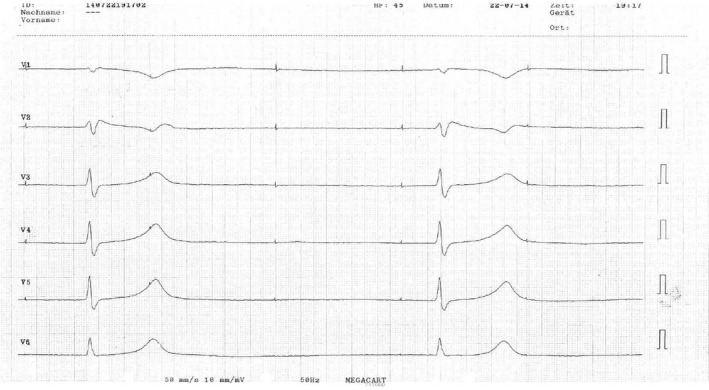
ECG during device interrogation (AAI pacing 60 bpm, atrial exit block) shows intrinsic heart rate of <25 bpm also with signs of hyperkalemia, but a different QRS morphology when compared to ECG 1.

**Figure 3:**
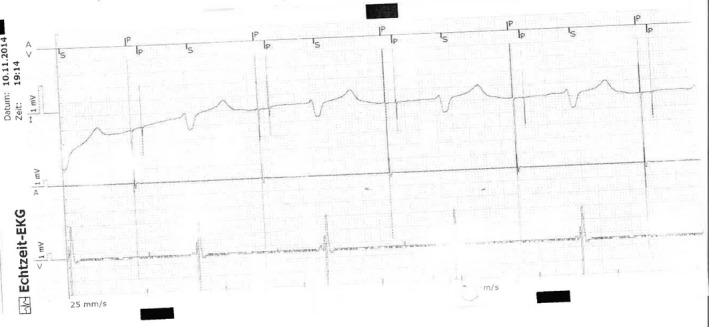
Electrogram upon device interrogation shows, from top to bottom, marker annotation, lead I of the surface ECG, and atrial (A) and ventricular (V) electrograms. Atrial and ventricular pacing is delivered, followed by a constantly coupled, but delayed ventricular sensing. In the atrial electrogram, no depolarization can be detected. From ventricular sensing, the next ventricular pacing is delivered after 1000 ms, corresponding to the programmed stimulation rate of 60 bpm.

This case illustrates the significant effects of hyperkalemia on patients with cardiac rhythm devices, which may include a rise in pacing thresholds, atrial and (possibly to a less extent) ventricular undersensing due to attenuation of the signals for sensing, T‐wave oversensing in recipients of implantable cardioverter‐defibrillators, and an increase in latency (delay from pacing stimulus to the onset of ventricular depolarization) 3. The latency of 600 ms in the presented case is extraordinarily long and – to our knowledge – not previously described. It is likely that faster pacing rate would have transformed first‐degree pacemaker exit block in more severe forms, also dependent on the programmed output 3. Hyperkalemia is frequent, especially in older patients undergoing intensive treatment for heart failure with potassium‐retaining drugs (and co‐existing renal failure). In the presented case, hyperkalemia was associated with a special form of treatment for a neuroendocrine tumor. Even if frequently encountered during this type of therapy, from an electrophysiological point of view, a high rate of suspicion is necessary not to overlook hyperkalemia as the reason for ventricular exit block. In fact, in this case, the typical signs of hyperkalemia in the surface ECG were the initial clue for correct diagnosis, treatment, and omission of surgical lead revision.

## Authorship

SA: contributed to patient care and manuscript draft, revision, and approval; FS: contributed to patient care, manuscript revision and approval, and data acquisition; JR: contributed to patient care, manuscript revision and approval, and data acquisition; CB: contributed to patient care and manuscript revision and approval; CL: contributed to patient care, manuscript revision and approval, and data acquisition.

## Conflict of Interest

None declared.
